# AI-enabled detection of QRS fragmentation from 12-lead electrocardiogram and its clinical relevance for predicting malignant arrhythmia onset

**DOI:** 10.3389/fcvm.2024.1464303

**Published:** 2024-10-21

**Authors:** Sebastian Ingelaere, Amalia Villa, Carolina Varon, Sabine Van Huffel, Bert Vandenberk, Rik Willems

**Affiliations:** ^1^ Department of Cardiovascular Sciences, Faculty of Medicine, KU Leuven, Leuven, Belgium; ^2^ Department of Cardiovascular Diseases, University Hospitals Leuven, Leuven, Belgium; ^3^ Stadius Centre for Dynamical Systems, Signal Processing and Data Analytics, Department of Electrical Engineering, Faculty of Engineering Sciences, KU Leuven, Leuven, Belgium

**Keywords:** QRS fragmentation, implantable-cardioverter defibrillator, sudden cardiac death, ventricular arrhythmias, artificial intelligence

## Abstract

**Background:**

Electrocardiographic markers differentiating between death caused by ventricular arrhythmias and non-arrhythmic death could improve the selection of patients for implantable cardioverter-defibrillator (ICD) implantation. QRS fragmentation (fQRS) is a parameter of interest, but subject to debate. We investigated the association of an automatically quantified probability of fragmentation with the outcome in ICD patients.

**Methods:**

From a single-center retrospective registry, all patients implanted with an ICD between January 1996 and December 2018 were eligible for inclusion. Patients with active pacing were excluded. From the electronical medical record, clinical characteristics at implantation were collected and a 12-lead ECG was exported and analyzed by a previously validated machine-learning algorithm to quantify the probability of fQRS. To compare fQRS(+) and fQRS(−) patients, dichotomization was performed using the Youden index. Patients with a high probability of fragmentation in any region (anterior, inferior or lateral), were labeled fQRS(+). The impact of this fQRS probability on outcomes was investigated using Cox regression.

**Results:**

A total of 1,242 patients with a mean age of 62.6 ± 11.5 years and a reduced left ventricular ejection fraction of 31 ± 12% were included of which 227 (18.3%) were female. The vast majority suffered from ischemic heart disease (64.3%) and were implanted in primary prevention (63.8%). 538 (43.3%) had a high probability of fragmentation in any region. Patients with a high probability of fragmentation had more frequently dilated cardiomyopathy (39.4% vs. 33.0%, *p* = 0.019), left bundle branch block (40.8% vs. 32.5%, *p* = 0.006) and a higher use of cardiac resynchronization therapy with defibrillator (CRT-D) devices (33.9% vs. 26.3%, *p* = 0.004). After adjustment in a multivariable Cox model, there was no significant association between the probability of global or regional fQRS and appropriate ICD therapy, inappropriate shock and short- or long-term mortality.

**Conclusion:**

There was no association between the automatically quantified probability of the presence of fQRS and outcome. This lack of predictive value might be due to the algorithm used, which identifies only the presence but not the severity of fragmentation.

## Introduction

Cardiovascular mortality remains one of the leading causes of death worldwide. Fatal cardiac events can be classified by their underlying mechanism; either mechanical based on pump failure, or electrical secondary to ventricular arrhythmias. While drug therapy, with or without resynchronization pacing, is pivotal in preventing progression to pump failure, implantation of an implantable cardioverter-defibrillator (ICD) stands as a cornerstone in the prevention of sudden death due to ventricular arrhythmias (1). Despite the significantly increased risk of life-threatening ventricular arrhythmias in patients with heart failure, that are candidates for ICD implantation in primary prevention, the majority of patients with an ICD do not experience life-saving interventions from their device (2–4). In the current era of improved medical heart failure treatment and possible declining benefit of ICD therapy, optimal risk stratification is crucial to select patients at high-risk for ventricular arrhythmias but at low risk of non-arrhythmic death (5).

The electrocardiogram (ECG) is a widely available, easy-to-perform, low-cost and low-risk diagnostic tool essential in cardiology practice with potential to refine arrhythmia risk stratification. The QRS complex represents the ventricular depolarization on ECG. Myocardial scarring results in heterogeneous myocardial activation, causing conduction delay and creating substrates for reentry circuits. Fragmentation of the QRS complex was initially described by Flowers et al. in 1969 and implies a heterogeneous activation pattern (6). Despite its potential, consensus on the prognostic value of fragmented QRS (fQRS) remains elusive in literature, partly due to its typical binary use based on visual estimation, which is prone to significant inter- and intra-observer variability (7). We recently developed an algorithm to automatically quantify the probability of the presence of QRS fragmentation (8). In this study we aim to investigate the relationship between this probability of the presence of QRS fragmentation and outcomes of post-ICD implantation.

## Methods

### Patient selection

All patients with ischemic or dilated cardiomyopathy, who received their first ICD device at the University Hospitals of Leuven between 01/01/1996 and 31/12/2018 were eligible for inclusion (9). Both primary and secondary prevention ICD indications were included. Single and dual chamber ICD devices (VVI/DDD) as well as cardiac resynchronization defibrillator devices (CRT-D) were included. Patients with ventricular pacing on the ECG at time of implantation were excluded. Analysis on this retrospective dataset was approved by the local ethical committee of the University Hospitals of Leuven (S56074).

### Quantification of QRS fragmentation

Pre-procedural resting 12-lead ECGs were recorded (paper speed of 25 mm/s, voltage of 10 mm/mV, sampling rate 250 Hz, filter range 0.05–150 Hz with notch filter at 50/60 Hz) and analyzed using the “GE Marquette 12SL™ ECG Analysis Program” (GE Medical Systems, Menomonee Falls, WI, USA). Raw ECG data were exported for QRS fragmentation analysis. Recently, we reported on an AI-enabled supervised machine learning algorithm designed to quantify the probability of QRS fragmentation (8). This method employs a novel and robust QRS segmentation strategy combining information from multiple leads to ensure the inclusion of critical Q and S wave information while excluding artifactual oscillations and abnormal heartbeats. A dataset of pre-ICD implantation ECGs from 673 patients implanted at UZ Leuven was used (including both narrow and broad QRS complexes). Patients were on average 62.4 ± 11.6 years old and had a severely reduced left ventricular ejection fraction (LVEF) of 32.3 ± 12.3%. The majority of patients were male (84.7%) and suffered from ischemic heart disease (66.3%). At the time of implantation, 57 patients (8.5%) were in atrial fibrillation, while the remaining ECGs exhibited sinus rhythm. These ECG recordings were evaluated lead-by-lead by five independent clinicians for presence or absence of QRS fragmentation using binary labeling. The sum of the scores per lead represented the level of agreement with signals scoring ′0′ (full agreement that the signal was non-fragmented) and ′5′ (full agreement that the signal was fragmented) used as the ground truth to train the model. Ten features as proposed by Goovaerts et al. were extracted from each signal (10). Three of these features were derived from phase-rectified signal averaging (PRSA) curves, which can detect oscillations in the QRS complex (mean derivative of the PRSA curve, slope and y-axis crossing of its linear fit). Six additional parameters were derived using variational mode decomposition (VMD), which decomposes signals into their frequency components. Lastly, the number of peaks within the QRS complex on the ECG was used. These ten features were used to train a Support Vector Machine (SVM), a machine learning classifier, to distinguish between fragmented and non-fragmented signals. Platt scaling was applied to convert the binary score into a continuous score by fitting a logistic regression, thus providing a probability of fragmentation. We used the UZ Leuven algorithm given the best performance in the UZ Leuven dataset (sensitivity 0.76, specificity 0.92, kappa 0.71). Output consisted of a continuous value between 0 and 1 for each signal, representing the probability of fragmentation. Mean values were calculated for anterior (V1–V5), lateral (I, aVL, V6) and inferior (II, III, aVF) regions.

### Endpoints

Patients in the registry were followed every 6–12 months. Device related endpoints were 1-year, 3-year and overall appropriate therapy (i.e., anti-tachypacing and shock) or appropriate shock and ever inappropriate shock-rate. ICD interventions were analyzed by the treating cardiologist and registered in a standardized manner in the electronic medical record. Mortality related endpoints were 1-year, 3-year and overall mortality as well as ICD resistant mortality (ICD-RM). Guidelines recommend to reserve ICD therapy to patients with an expectation of good-quality survival of more than 1 year. Therefore, an ICD was considered clinically useful when the ICD provided appropriate therapy and the patient survived implantation by 1 year and appropriate shock by 30 days. As such, to identify the subgroup of patients for whom ICD implantation proved futile, ICD-RM was defined as death within the first year after ICD implantation or within 30 days after a first shock or if death occurred without any documented appropriate ICD intervention during follow-up (4). Mortality was deducted from the national population register, linked to the electronic medical record, ensuring completeness of data. In case of heart transplantation, date of transplantation was considered last date of follow-up.

### Statistical analysis

Categorical variables are presented as number with percentages and continuous variables as mean with standard deviation. Based on the receiver operating characteristic curve, cut-off for fragmentation was set at 0.642, the value with the highest Youden index (0.651) with at least 95% specificity, as we want to include true fragmentation. Patients with or without fragmentation in any region were compared. We compared continuous variables by a Mann-Whitney *U* test and categorical variables by a Chi² test. *P*-values ≤ 0.05 were considered significant. Multicollinearity was tested by calculating the variance of inflation factors (VIFs). Univariate Cox regression analysis was performed to identify predictors of the predefined endpoints. For each endpoint, a multivariable Cox regression analysis was performed using the ENTER method with selection of variables based on a univariable *p*-value < 0.100. Statistical analysis was performed using SPSS Statistics (version 29, IBM Corp., Armonk, NY, USA).

## Results

A total of 1,242 patients were included, with their baseline characteristics presented in Table 1. Almost half of them had QRS fragmentation in any region (*N* = 538, 43.3%). When comparing patients with or without QRS fragmentation at implantation, no significant disparities were observed. The average age of the cohort was 62.6 ± 11.5 years, without significant difference between patients with or without fragmentation. LVEF was comparable across patients with QRS fragmentation (30 ± 11%) as without fragmentation (31 ± 11%). Arterial hypertension (*N* = 665, 53.5%) and atrial fibrillation (*N* = 355, 28.6%) were the most common comorbidities with balanced prevalence between both groups. Only a minority of the included patients were female (*N* = 227, 18.3%). The sole noteworthy distinctions were a lower incidence of ischemic heart disease in patients with QRS fragmentation compared to those with dilated cardiomyopathy (60.6% vs. 67.0%, *p* = 0.019), a higher prevalence of CRT-D usage (33.9% vs. 26.3%, *p* = 0.004), and a greater frequency of LBBB (40.8% vs. 32.5%, *p* = 0.006).

**Table 1:** Baseline patient characteristics by QRS fragmentation.

QRS fragmentation	Overall		fQRS (−)		fQRS (+)		*p*-value
*N* = 1,242	Mean	SD	Mean	SD	Mean	SD	
Age (years)	62.6	11.5	62.9	10.6	61.1	12.9	0.165
BMI (kg/m²)	26.4	4.4	26.6	4.4	26.6	4.3	0.856
LVEF (%)	31	12	31	11	30	11	0.425
Creatinine (mg/dl)	1.26	0.48	1.24	0.48	1.24	0.45	0.628
QTc (ms)	443	51	444	50	443	52	0.887
	Number	%	Number	%	Number	%	
Female	227	18.3	121	17.2	106	19.7	0.256
IHD (vs. DCM)	798	64.3	472	67.0	326	60.6	**0** **.** **019**
Secondary (vs. primary)	450	36.2	256	36.4	194	36.1	0.912
NYHA							
I	323	26.0	186	26.4	137	25.5	0.358
II	551	44.4	324	46.0	228	42.4	
III	359	28.9	190	27.0	169	31.4	
IV	8	0.6	4	0.6	4	0.7	
CRT-D (vs. VVI/DDD)	367	29.6	185	26.3	182	33.9	**0**.**004**
Stroke	134	10.8	72	10.2	62	11.5	0.465
DM	261	21.0	149	21.2	112	20.8	0.882
AF	355	28.6	192	27.3	163	30.4	0.234
AHT	665	53.5	390	55.4	275	51.1	0.134
BB	1,058	85.3	600	85.3	458	85.1	0.914
ACE-I or ARB	1,091	87.9	625	88.9	466	86.6	0.221
Loop	606	48.8	334	47.5	272	50.6	0.287
Aldactone	682	55.0	370	52.6	312	58.0	0.060
Anti-aggregant	748	60.3	439	62.4	309	57.4	0.074
Anticoagulation	420	33.8	226	32.1	194	36.1	0.144
Amiodarone	442	35.6	248	35.3	194	36.1	0.776
Sotalol	11	0.9	8	1.1	3	0.6	0.280
Digitalis	106	8.5	61	8.7	45	8.4	0.845
Statin	759	61.2	442	62.9	317	58.9	0.157
Ever appropriate	435	35.0	256	36.4	179	33.3	0.258
LBBB	386	36.2	192	32.5	194	40.8	**0**.**006**

Baseline patient characteristics with comparison between patients with [fQRS(+)] or without [fQRS(−)]. Age (years); body mass index, BMI (kg/m²); left ventricular ejection fraction, LVEF (%); creatinine (mg/dL); QTc interval, QTc (ms); ischemic heart disease, IHD; dilated cardiomyopathy, DCM; secondary vs. primary prevention indication; New York Heart Association class, NYHA; diabetes mellitus, DM; atrial fibrillation, AF; arterial hypertension, AHT; betablocker, BB; angiotensin converting enzyme inhibitor, ACE-I; angiotensin receptor blocker, ARB; loop diuretic, loop; ever appropriate ICD therapy (anti-tachypacing or shock), ever appropriate; left bundle branch block, LBBB; CRT-D, cardiac resynchronisation therapy with defibrillator; VVI/DDD, single and dual chamber ICD devices.

Significant *p*-values in bold.

### QRS fragmentation and mortality

**Figure 1:**
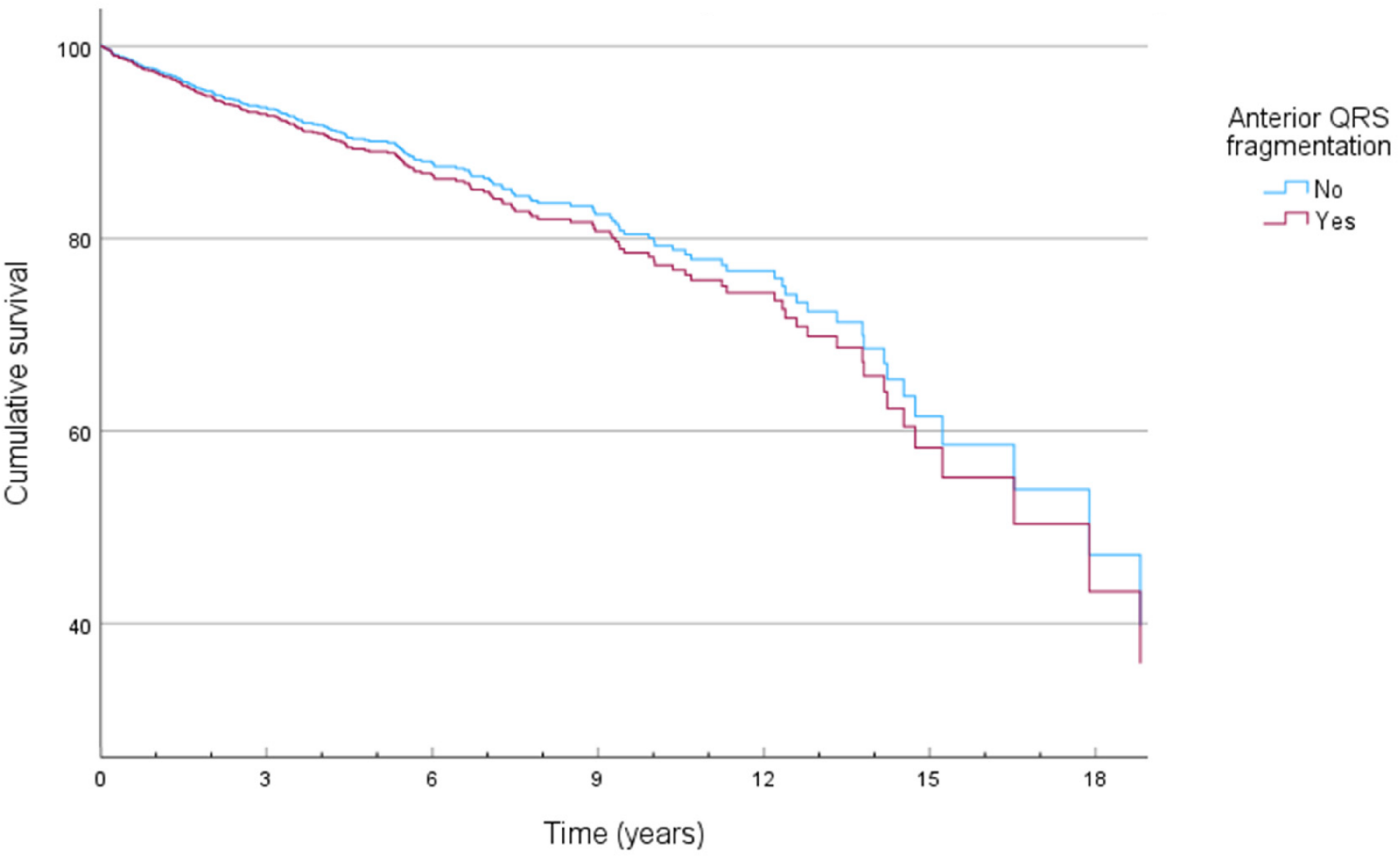
Cox-derived survival plot of ICD resistant mortality (ICD-RM) stratified by the presence of anterior QRS fragmentation.

**Figure 2:**
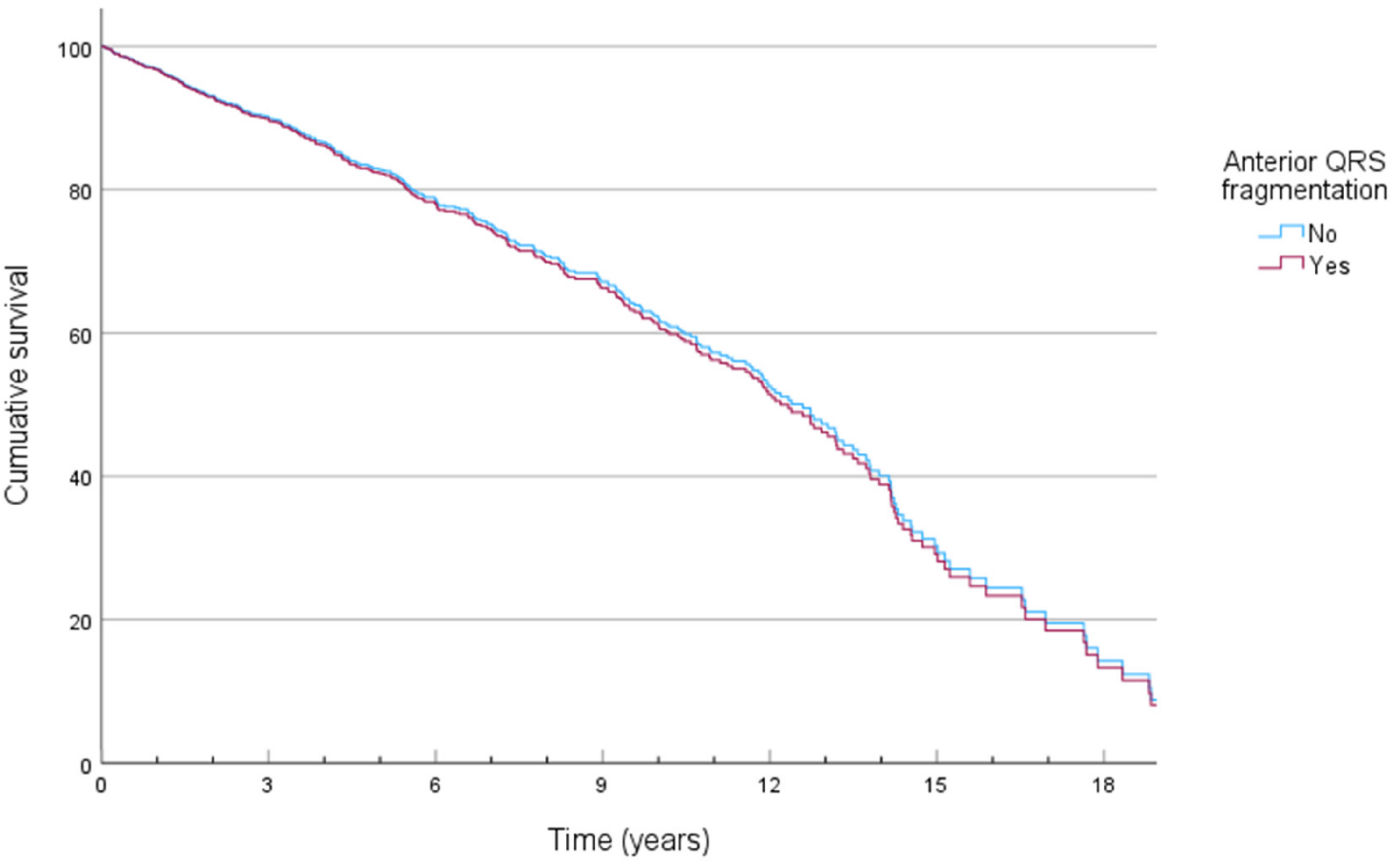
Cox-derived survival plot of overall mortality stratified by the presence of anterior QRS fragmentation.

QRS fragmentation showed no predictive value for 1-year nor 3-year survival by univariate Cox regression analysis (Supplementary Appendix Tables S1–S2). Anterior region fragmentation appeared to predict ICD-RM (HR 1.668, *p* = 0.036) and approached significance for overall mortality (HR 1.467, *p* = 0.058) (Supplementary Appendix Tables S3–S4). Nonetheless, subsequent multivariable Cox modeling showed that anterior fragmentation lost its predictive power regarding ICD-RM (HR 1.112, *p* = 0.730). Significant predictors of ICD-RM were more pronounced heart failure symptoms, indicated by the detrimental impact of advanced NYHA status while there is a protective effect of CRT-D and better-preserved LVEF (Table 2). Cox-derived survival plot of ICD-RM stratified by anterior QRS fragmentation is presented in Figure 1. Overall mortality increased with age, higher creatinine, ischemic heart disease (IHD), worse NYHA status, most comorbidities and the use of loop diuretics and digitalis. A better preserved LVEF was associated with an improved overall survival (Table 3). Cox-derived survival plot of overall mortality stratified by anterior QRS fragmentation is presented in Figure 2.

**Table 2:** Multivariable Cox regression regarding ICD resistant mortality (ICD-RM).

ICD-RM	HR	95% Confidence interval		*p*-value
		Lower	Upper	
Age (/year)	1.061	1.042	1.081	**<0** **.** **001**
BMI (/kg/m²)	0.930	0.894	0.967	**<0**.**001**
LVEF (/%)	0.977	0.963	0.992	**0**.**003**
Creatinine (/mg/dl)	1.422	1.121	1.804	**0**.**004**
QTc (/ms)	1.001	0.998	1.003	0.673
Female	0.994	0.647	1.529	0.979
IHD (vs. DCM)	1.244	0.842	1.838	0.272
NYHA	ref			
I				
II	1.960	1.291	2.977	**0**.**002**
III	2.505	1.510	4.158	**<0**.**001**
IV	7.930	1.731	36.317	**0**.**008**
CRT-D (vs. VVI/DDD)	0.554	0.363	0.845	**0**.**006**
Stroke	1.274	0.807	2.009	0.298
DM	1.977	1.410	2.772	**<0**.**001**
AF	1.995	1.386	2.873	**<0**.**001**
AHT	0.930	0.681	1.269	0.646
Loop	1.535	1.094	2.153	**0**.**013**
Anticoagulation	0.836	0.580	1.207	0.340
Amiodarone	1.133	0.829	1.547	0.434
Statin	1.315	0.944	1.833	0.106
Anterior fQRS	1.112	0.609	2.029	0.730

Age (years); body mass index, BMI (kg/m²); left ventricular ejection fraction, LVEF (%); creatinine (mg/dL); QTc interval, QTc (ms); ischemic heart disease, IHD; dilated cardiomyopathy, DCM; New York Heart Association class, NYHA; diabetes mellitus, DM; atrial fibrillation, AF; arterial hypertension, AHT; loop diuretic, loop; CRT-D, cardiac resynchronisation therapy with defibrillator; VVI/DDD, single and dual chamber ICD devices.

Significant *p*-values in bold.

**Table 3:** Multivariable Cox regression regarding overall mortality.

Mortality (all)	HR	95% Confidence interval		*p*-value
		Lower	Upper	
Age (year)	1.050	1.036	1.063	**<0** **.** **001**
LVEF (/%)	0.979	0.968	0.989	**<0**.**001**
Creatinine (/mg/dl)	1.460	1.214	1.754	**<0**.**001**
QTc (/ms)	1.000	0.998	1.002	0.893
Female	0.939	0.680	1.295	0.699
IHD (vs. DCM)	1.536	1.173	2.012	**0**.**002**
Secondary (vs. primary)	1.240	0.966	1.590	0.091
NYHA	ref			
I				
II	1.602	1.220	2.103	**0**.**001**
III	1.461	1.054	2.027	**0**.**023**
IV	3.965	1.190	13.207	**0**.**025**
Stroke	1.400	1.007	1.947	**0**.**045**
DM	1.638	1.281	2.095	**<0**.**001**
AF	1.378	1.057	1.796	**0**.**018**
AHT	0.980	0.783	1.226	0.859
BB	0.833	0.640	1.084	0.174
Loop	1.396	1.096	1.778	**0**.**007**
Anticoagulation	0.931	0.713	1.215	0.598
Amiodarone	1.159	0.917	1.464	0.216
Digitalis	1.544	1.105	2.157	**0**.**011**
Anterior fQRS	1.034	0.655	1.631	0.887

Age (years); left ventricular ejection fraction, LVEF (%); creatinine (mg/dL); QTc interval, QTc (ms); ischemic heart disease, IHD; dilated cardiomyopathy, DCM; secondary vs. primary prevention indication; New York Heart Association class, NYHA; diabetes mellitus, DM; atrial fibrillation, AF; arterial hypertension, AHT; betablocker, BB; loop diuretic, loop.

Significant *p*-values in bold.

### QRS fragmentation and ICD therapy

In univariate Cox regression analysis, neither global nor regional QRS fragmentation exhibited any predictive value concerning clinically relevant device related endpoints, such as appropriate ICD intervention, appropriate ICD shock or inappropriate ICD shock (Supplementary Appendix Tables S5–S7). Secondary prevention and the use of digitalis were linked to an increased risk of appropriate ICD therapy, with or without shock (Supplementary Appendix Tables S8, S9). Conversely, patients treated with a statin exhibited a reduced risk of appropriate ICD shock (Supplementary Appendix Table S9). Even after adjustment, the presence of atrial fibrillation was associated with a higher likelihood of inappropriate ICD shocks (HR 1.992, *p* < 0.001). In contrast, older patients had a diminished risk of inappropriate ICD interventions (HR 0.981, *p* = 0.010) (Supplementary Appendix Table S10).

## Discussion

The presence of QRS fragmentation has been extensively studied in various cardiac conditions as a potential marker of adverse outcomes (11–14). The current analysis found no association between the automatically quantified probability of QRS fragmentation and patient outcomes.

### Prevalence of fQRS

The overall prevalence of fQRS in our cohort (43.3%) is higher compared to an analysis on MADIT II trial patients (33%) which might be due to differences in patient characteristics as the latter only included patients eligible for ICD in primary prevention (15). In a Finnish cohort of middle-aged subjects, the overall prevalence of fQRS was remarkably lower (19.7%). However, they included patients with and without cardiac antecedents (21.4% and 75.9% respectively) with a quasi-balanced cohort in terms of sex-category (52.3% men) and a notable lower average age (16). The same research group studied the prevalence of fQRS in patients without cardiac disease (19.0%) vs. ARTEMIS study patients with coronary artery disease with and without previous myocardial infarction (39.5% and 35.3% respectively) vs. SCD victims from the FinGesture study (53.8%) (17). Accounting for the inclusion of secondary prevention patients (considered as aborted SCD victims) and the preponderance of IHD in our cohort, we assume the prevalence of fQRS in our study being representative. Inferior fragmentation was the most prevalent in our cohort as well as in all aforementioned study populations (15–17). In contrast to findings of Engstrom et al., we did not observe a significant difference in QRS fragmentation with age, nor was there a predisposition to fragmentation in males compared to females or any significant difference in the prevalence of diabetes or atrial fibrillation (14).

### Predictive value of fQRS

In accordance with previous findings, there was no difference in NYHA functional status or the presence of ventricular arrhythmias, as defined by appropriate ICD therapy in patients with or without fQRS (18). The absence of significant predictive value of QRS fragmentation in this study contrasts with literature and diverges from our previous findings despite overlap in patients as this study is based on a single-center registry, updated until 2018 (19). Previous reports showed that the presence of fragmentation on ECG at hospital admission in acute STEMI patients was an independent predictor of in-hospital ventricular arrhythmias (20). Furthermore, a recent meta-analysis showed a significant prognostic value for fQRS in patients with acute myocardial infarction with an increased risk of in-hospital mortality and MACE, as well as long-term mortality and MACE (21). Another meta-analysis in heart failure patients also withheld fQRS as a predictor of ventricular arrhythmias but only when primary and secondary prevention patients were pooled as there was no significance in primary prevention alone (14). As patients with primary prevention ICD indications are most relevant for optimizing risk stratification, we performed a subgroup analysis but the results remained unchanged (Supplementary Appendix Table S11).

Further, our analysis did not establish an association between global nor regional QRS fragmentation and mortality. Terho et al. found an association between lateral QRS fragmentation and adverse survival rates in subjects with pre-existing cardiac diseases, while there was no association with mortality in healthy middle-aged subjects (17). Our findings contribute to the controversy surrounding fQRS. A meta-analysis by Rosengarten et al. found a significant association between fQRS and both all-cause mortality and SCD (22). However, more than half of the included studies were not significant with an important heterogeneity (*I* ² = 94%) resulting in a relative risk with broad 95% confidence interval [1.71 (1.02–2.85), *p* = 0.04] (22). The effect was mainly driven by two retrospective studies and one prospective study, the latter involving patients with higher LVEF, fewer comorbidities and only a small proportion of ICD recipients (23–25).

Despite the absence of predictive value of anterior QRS fragmentation concerning ICD-RM as well as overall mortality after correction for confounders, several clinical parameters can still predict survival after ICD implantation. As previous research of our group demonstrated that ICD-RM occurred in up to a quarter of all patients after 5 years, it is not surprising that there is a significant overlap between the predictors for ICD-RM and overall mortality (4). Aging is inherently associated with a higher risk of mortality, regardless of any ICD interventions. Parameters reflecting a generally worse clinical condition—such as lower LVEF, higher creatinine (indicating poorer renal function), higher NYHA functional class and comorbidities like diabetes and atrial fibrillation—logically predict both ICD-RM and overall mortality. It is of interest that the use of CRT-D was associated with lower ICD-RM, possibly because addressing pump failure, reduces heart failure-related mortality, creating a window of opportunity for ventricular arrhythmias to occur. Subsequently, these ventricular arrhythmias can be terminated by the ICD device. While cardiac scar in IHD is known to be an excellent substrate for the development of re-entry phenomena leading to malignant ventricular arrhythmias, which can be terminated by an ICD, it also contributes to pump dysfunction and heart failure-related mortality, which is not modulated by a conventional ICD.

To ensure the robustness of our analysis, we checked for multicollinearity by calculating VIFs and found no evidence of multicollinearity among the variables included in the multivariable analysis. Furthermore, by including all clinically relevant covariables, we avoided specification bias.

### Annotation and quantification of fQRS

Our findings should be interpreted with the limitations of visually scored QRS fragmentation in mind. Previous work of our group highlighted the inter- and intra-observer variability in interpretation of QRS fragmentation (7), prompting this current study aimed to assess the predictive value of QRS fragmentation in patients implanted with an ICD using an automated machine-learning algorithm (8). As our algorithm was trained with signals that were annotated by clinicians, it encompasses the aforementioned inter- and intra-observer variability. Furthermore, our model is trained with only ten preselected features, thus excluding other potential predictive features of the ECG signals. Therefore, in an effort to detect and quantify QRS fragmentation in a standardized and automated manner, the proposed algorithm actually estimates the likelihood that a clinician would perceive an ECG signal as fragmented based on 5 independent observers applying the Das criteria (8, 23, 26). Note that the original criteria proposed by Das et al. only distinguish between the presence or absence of fragmentation without assigning a numerical score reflecting the severity of fragmentation (26). To date, actual quantification of QRS fragmentation remains a challenge. Roudijk et al. attempted to quantify QRS fragmentation by scoring each deflection in the QRS complexes of 12 leads and found a significant association between higher scores and the presence of arrhythmogenic right ventricular cardiomyopathy, without being able to predict ventricular arrhythmias or SCD (27). In general, the results of visual QRS fragmentation should be interpreted with caution as the traditional visual ECG interpretation is inherently limited by subjectivity and observer variability emphasizing the importance of clear criteria to distinguish fragmentation due to myocardial scar from benign normal variants (7, 28). Therefore, computational quantification is increasingly pursued. Iglesias et al. approached this challenge using continuous wavelet transformation focusing on the frequency-domain instead of time-domain analysis (29). Another automated ECG-based evaluation of myocardial scar, the Selvester score, was significantly associated with mortality and device-related endpoints (30). However, the question arises whether all of these approaches identify fragmentation as described by Das or enclose other information. The lack of a uniform methodological approach compromises the reproducibility of studies in this field. Additionally, using data from 12 leads separately increases the risk of statistical errors. The latter advocates for dimensionality reduction, which reduces the risk of type I error by multiple testing and has the potential to enhance the signal-to-noise ratio. Recently, QRS micro-fragmentation was presented as a promising predictor of mortality (31). This analysis overcomes these hurdles as the analysis uses singular value decomposition of the QRS complex to project the information of all QRS complexes into optimized three perpendicular dimensions. Subsequently, the ECG is reconstructed back from this three-dimensional projection and micro-fragmentation is quantified as the difference between the original and reconstructed ECG signals. By quantifying micro-fragmentation as percentage, it presents as a single, weighted value no longer prone to multicollinearity between different leads.

### Limitations

All patients in this study were implanted in a tertiary hospital, which may introduce referral bias. Due to the design, general limitations inherent to retrospective, non-randomized research performed in a single center are valid. We only assessed the ECG at implantation not taking into account possible dynamical changes over time. Although we have reliable mortality data, we are blinded to the underlying mechanism of death while differentiation between arrhythmic and non-arrhythmic death might provide useful insights. Future work should focus on the true quantification of QRS fragmentation. As an extension of this research, improved prediction of outcomes might be possible by training new machine-learning models linking the current features directly to the outcome parameters, without the intermediate step of fQRS detection.

## Conclusion

There was no association between the automatically quantified probability of QRS fragmentation and outcome when correcting for confounders by Cox proportional hazards modeling. While the algorithm quantifies the probability of QRS fragmentation, it does not directly assess the risk of arrhythmia or other adverse events. Instead, it uses fragmentation as an intermediate index, which might compromise its predictive value. Further research is warranted to improve quantification techniques and to establish a more robust understanding of the clinical relevance of fQRS in ICD patients.

## Data Availability

The raw data supporting the conclusions of this article will be made available by the authors, without undue reservation.
