# Supplementary material for: AI-enabled detection of QRS fragmentation from 12-lead electrocardiogram and its clinical relevance for predicting malignant arrhythmia onset
Source: Front Cardiovasc Med. 2024 Oct 21;11:1464303. doi: 10.3389/fcvm.2024.1464303 (PMC11532058; doi:10.3389/fcvm.2024.1464303)
Supplement: Supplementary file 1 [file Datasheet1.docx]

# Supplemental appendix fQRS

Table S1. Univariate Cox analysis regarding 1-year mortality

| **1-YEAR MORTALITY** |  | 95% confidence interval | |  |
| --- | --- | --- | --- | --- |
|  | HR | lower | Upper | p-value |
| Age (/year) | 1.051 | 1.023 | 1.080 | **<0.001** |
| BMI (/kg/m²) | 0.904 | 0.848 | 0.964 | **0.002** |
| LVEF (/%) | 1.000 | 0.979 | 1.022 | 0.992 |
| Creatinin (/mg/dL) | 1.819 | 1.394 | 2.374 | **<0.001** |
| QTc (/ms) | 1.001 | 0.996 | 1.007 | 0.632 |
| Female | 1.265 | 0.699 | 2.288 | 0.437 |
| IHD (vs DCM) | 3.486 | 1.723 | 7.053 | **0.001** |
| Secondary (vs primary) | 2.178 | 1.332 | 3.564 | **0.002** |
| NYHA I | ref |  |  |  |
| NYHA II | 1.646 | 0.768 | 3.526 | 0.200 |
| NYHA III | 2.985 | 1.413 | 6.306 | **0.004** |
| NYHA IV | 4.821 | 0.611 | 38.057 | 0.136 |
| CRT-D (vs VVI/DDD) | 1.088 | 0.641 | 1.846 | 0.754 |
| Stroke | 2.203 | 1.198 | 4.051 | **0.011** |
| DM | 2.033 | 1.214 | 3.406 | **0.007** |
| AF | 2.722 | 1.667 | 4.445 | **<0.001** |
| AHT | 1.461 | 0.881 | 2.423 | 0.142 |
| BB | 0.831 | 0.434 | 1.590 | 0.576 |
| ACE-I or ARB | 0.433 | 0.243 | 0.773 | **0.005** |
| Loop | 2.192 | 1.301 | 3.693 | **0.003** |
| Aldactone | 0.811 | 0.497 | 1.323 | 0.401 |
| Anti-aggregant | 1.034 | 0.626 | 1.709 | 0.896 |
| Anticoagulation | 1.544 | 0.942 | 2.529 | 0.085 |
| Amiodarone | 2.376 | 1.450 | 3.894 | **0.001** |
| Sotalol | 0.049 | 0.000 | 5067.196 | 0.609 |
| Digitalis | 1.104 | 0.476 | 2.558 | 0.818 |
| Statin | 0.764 | 0.467 | 1.250 | 0.284 |
| LBBB | 0.936 | 0.520 | 1.686 | 0.826 |
| Anterior fQRS | 1.517 | 0.609 | 3.781 | 0.371 |
| Lateral fQRS | 1.502 | 0.842 | 2.678 | 0.168 |
| Inferior fQRS | 0.740 | 0.436 | 1.255 | 0.264 |
| Any fQRS | 0.836 | 0.506 | 1.381 | 0.485 |

Age (years); body mass index, BMI (kg/m²); left ventricular ejection fraction, LVEF (%); creatinin (mg/dL); QTc interval, QTc (ms); ischemic heart disease, IHD; dilated cardiomyopathy, DCM; secondary vs primary prevention indication; New York Heart Association class, NYHA; diabetes mellitus, DM; atrial fibrillation, AF; arterial hypertension, AHT; betablocker, BB; angiotensin converting enzyme inhibitor, ACE-I; angiotensin receptor blocker, ARB; loop diuretic, loop; ever appropriate ICD therapy (anti-tachypacing or shock), ever appropriate; left bundle branch block, LBBB.

Table S2. Univariate Cox analysis regarding 3-year mortality

| **3-YEAR MORTALITY** |  | 95% confidence interval | |  |
| --- | --- | --- | --- | --- |
|  | HR | lower | Upper | p-value |
| Age (/year) | 1.052 | 1.035 | 1.070 | **<0.001** |
| BMI (/kg/m²) | 0.952 | 0.918 | 0.988 | **0.010** |
| LVEF (/%) | 0.990 | 0.977 | 1.004 | 0.172 |
| Creatinin (/mg/dL) | 1.874 | 1.604 | 2.190 | **<0.001** |
| QTc (/ms) | 1.002 | 0.999 | 1.006 | 0.165 |
| Female | 0.862 | 0.571 | 1.302 | 0.481 |
| IHD (vs DCM) | 2.230 | 1.537 | 3.237 | **<0.001** |
| Secondary (vs primary) | 1.854 | 1.370 | 2.509 | **<0.001** |
| NYHA I | ref |  |  |  |
| NYHA II | 2.096 | 1.317 | 3.334 | **0.002** |
| NYHA III | 2.637 | 1.636 | 4.251 | **<0.001** |
| NYHA IV | 6.231 | 1.871 | 20.754 | **0.003** |
| CRT-D (vs VVI/DDD) | 0.979 | 0.702 | 1.365 | 0.900 |
| Stroke | 1.748 | 1.165 | 2.623 | **0.007** |
| DM | 1.780 | 1.283 | 2.469 | **0.001** |
| AF | 2.113 | 1.558 | 2.865 | **<0.001** |
| AHT | 1.376 | 1.010 | 1.875 | **0.043** |
| BB | 0.748 | 0.506 | 1.104 | 0.144 |
| ACE-I or ARB | 0.883 | 0.564 | 1.383 | 0.588 |
| Loop | 1.863 | 1.362 | 2.549 | **<0.001** |
| Aldactone | 0.804 | 0.595 | 1.089 | 0.159 |
| Anti-aggregant | 1.019 | 0.748 | 1.389 | 0.905 |
| Anticoagulation | 1.376 | 1.011 | 1.872 | **0.042** |
| Amiodarone | 2.162 | 1.597 | 2.927 | **<0.001** |
| Sotalol | 0.640 | 0.090 | 4.567 | 0.656 |
| Digitalis | 1.571 | 0.994 | 2.481 | 0.053 |
| Statin | 0.929 | 0.682 | 1.264 | 0.638 |
| LBBB | 1.205 | 0.859 | 1.691 | 0.281 |
| Anterior fQRS | 1.269 | 0.689 | 2.339 | 0.445 |
| Lateral fQRS | 1.414 | 0.982 | 2.037 | 0.063 |
| Inferior fQRS | 0.899 | 0.656 | 1.233 | 0.509 |
| Any fQRS | 1.028 | 0.758 | 1.394 | 0.860 |

Age (years); body mass index, BMI (kg/m²); left ventricular ejection fraction, LVEF (%); creatinin (mg/dL); QTc interval, QTc (ms); ischemic heart disease, IHD; dilated cardiomyopathy, DCM; secondary vs primary prevention indication; New York Heart Association class, NYHA; diabetes mellitus, DM; atrial fibrillation, AF; arterial hypertension, AHT; betablocker, BB; angiotensin converting enzyme inhibitor, ACE-I; angiotensin receptor blocker, ARB; loop diuretic, loop; ever appropriate ICD therapy (anti-tachypacing or shock), ever appropriate; left bundle branch block, LBBB.

Table S3. Univariate Cox analysis regarding ICD resistant mortality (ICD-RM)

| **ICD-RM** |  | 95% confidence interval | |  |
| --- | --- | --- | --- | --- |
|  | HR | Lower | Upper | p-value |
| Age (/year) | 1.066 | 1.051 | 1.081 | **<0.001** |
| BMI (/kg/m²) | 0.971 | 0.943 | 1.000 | **0.049** |
| LVEF (/%) | 0.988 | 0.978 | 0.998 | **0.018** |
| Creatinin (/mg/dL) | 1.923 | 1.681 | 2.199 | **<0.001** |
| QTc (/ms) | 1.002 | 1.000 | 1.005 | 0.066 |
| Female | 0.746 | 0.528 | 1.055 | 0.097 |
| IHD (vs DCM) | 1.932 | 1.449 | 2.577 | **<0.001** |
| Secondary (vs primary) | 1.174 | 0.924 | 1.492 | 0.189 |
| NYHA I | ref |  |  |  |
| NYHA II | 1.813 | 1.305 | 2.520 | **<0.001** |
| NYHA III | 2.650 | 1.886 | 3.723 | **<0.001** |
| NYHA IV | 4.278 | 1.036 | 17.664 | **0.045** |
| CRT-D (vs VVI/DDD) | 1.243 | 0.959 | 1.609 | 0.100 |
| Stroke | 1.534 | 1.075 | 2.190 | **0.018** |
| DM | 1.883 | 1.446 | 2.453 | **<0.001** |
| AF | 2.361 | 1.862 | 2.994 | **<0.001** |
| AHT | 1.530 | 1.200 | 1.950 | **0.001** |
| BB | 0.944 | 0.699 | 1.276 | 0.707 |
| ACE-I or ARB | 0.872 | 0.619 | 1.227 | 0.432 |
| Loop | 1.960 | 1.536 | 2.501 | **<0.001** |
| Aldactone | 1.068 | 0.841 | 1.357 | 0.590 |
| Anti-aggregant | 1.063 | 0.828 | 1.366 | 0.630 |
| Anticoagulation | 1.653 | 1.297 | 2.106 | **<0.001** |
| Amiodarone | 1.562 | 1.231 | 1.981 | **<0.001** |
| Sotalol | 1.471 | 0.471 | 4.594 | 0.507 |
| Digitalis | 1.147 | 0.760 | 1.731 | 0.512 |
| Statin | 1.312 | 1.026 | 1.678 | **0.030** |
| LBBB | 1.168 | 0.887 | 1.538 | 0.268 |
| Anterior fQRS | 1.668 | 1.033 | 2.692 | **0.036** |
| Lateral fQRS | 1.277 | 0.939 | 1.735 | 0.119 |
| Inferior fQRS | 0.918 | 0.716 | 1.178 | 0.502 |
| Any fQRS | 0.953 | 0.749 | 1.213 | 0.698 |

Age (years); body mass index, BMI (kg/m²); left ventricular ejection fraction, LVEF (%); creatinin (mg/dL); QTc interval, QTc (ms); ischemic heart disease, IHD; dilated cardiomyopathy, DCM; secondary vs primary prevention indication; New York Heart Association class, NYHA; diabetes mellitus, DM; atrial fibrillation, AF; arterial hypertension, AHT; betablocker, BB; angiotensin converting enzyme inhibitor, ACE-I; angiotensin receptor blocker, ARB; loop diuretic, loop; ever appropriate ICD therapy (anti-tachypacing or shock), ever appropriate; left bundle branch block, LBBB.

Table S4. Univariate Cox analysis regarding overall mortality

| **OVERALL MORTALITY** |  | 95% confidence interval | |  |
| --- | --- | --- | --- | --- |
|  | HR | lower | Upper | p-value |
| Age (/year) | 1.061 | 1.050 | 1.072 | **<0.001** |
| BMI (/kg/m²) | 0.998 | 0.977 | 1.020 | 0.860 |
| LVEF (/%) | 0.988 | 0.981 | 0.996 | **0.002** |
| Creatinin (/mg/dL) | 1.863 | 1.662 | 2.089 | **<0.001** |
| QTc (/ms) | 1.002 | 1.000 | 1.004 | **0.035** |
| Female | 0.733 | 0.562 | 0.955 | **0.022** |
| IHD (vs DCM) | 1.849 | 1.487 | 2.297 | **<0.001** |
| Secondary (vs primary) | 1.286 | 1.072 | 1.542 | **0.007** |
| NYHA I | ref |  |  |  |
| NYHA II | 1.717 | 1.361 | 2.167 | **<0.001** |
| NYHA III | 2.090 | 1.623 | 2.690 | **<0.001** |
| NYHA IV | 4.128 | 1.304 | 13.069 | **0.016** |
| CRT-D (vs VVI/DDD) | 1.093 | 0.890 | 1.343 | 0.397 |
| Stroke | 1.746 | 1.340 | 2.275 | **<0.001** |
| DM | 1.781 | 1.448 | 2.191 | **<0.001** |
| AF | 1.871 | 1.555 | 2.250 | **<0.001** |
| AHT | 1.576 | 1.311 | 1.894 | **<0.001** |
| BB | 0.762 | 0.617 | 0.940 | **0.011** |
| ACE-I or ARB | 1.025 | 0.784 | 1.341 | 0.857 |
| Loop | 1.829 | 1.523 | 2.198 | **<0.001** |
| Aldactone | 1.106 | 0.922 | 1.326 | 0.280 |
| Anti-aggregant | 1.047 | 0.865 | 1.267 | 0.638 |
| Anticoagulation | 1.436 | 1.189 | 1.735 | **<0.001** |
| Amiodarone | 1.553 | 1.296 | 1.861 | **<0.001** |
| Sotalol | 1.267 | 0.473 | 3.393 | 0.638 |
| Digitalis | 1.592 | 1.207 | 2.100 | **0.001** |
| Statin | 1.108 | 0.922 | 1.330 | 0.273 |
| LBBB | 1.153 | 0.935 | 1.422 | 0.184 |
| Anterior fQRS | 1.467 | 0.988 | 2.180 | 0.058 |
| Lateral fQRS | 1.083 | 0.846 | 1.386 | 0.525 |
| Inferior fQRS | 0.987 | 0.819 | 1.190 | 0.891 |
| Any fQRS | 0.998 | 0.832 | 1.197 | 0.984 |

Age (years); body mass index, BMI (kg/m²); left ventricular ejection fraction, LVEF (%); creatinin (mg/dL); QTc interval, QTc (ms); ischemic heart disease, IHD; dilated cardiomyopathy, DCM; secondary vs primary prevention indication; New York Heart Association class, NYHA; diabetes mellitus, DM; atrial fibrillation, AF; arterial hypertension, AHT; betablocker, BB; angiotensin converting enzyme inhibitor, ACE-I; angiotensin receptor blocker, ARB; loop diuretic, loop; ever appropriate ICD therapy (anti-tachypacing or shock), ever appropriate; left bundle branch block, LBBB.

Table S5. Univariate Cox analysis regarding appropriate ICD therapy

| **EVER APPROPRIATE THERAPY** |  | 95% confidence interval | |  |
| --- | --- | --- | --- | --- |
|  | HR | Lower | Upper | p-value |
| Age (/year) | 1.000 | 0.991 | 1.008 | 0.963 |
| BMI (/kg/m²) | 0.998 | 0.976 | 1.020 | 0.868 |
| LVEF (/%) | 1.001 | 0.994 | 1.009 | 0.712 |
| Creatinine (/mg/dL) | 1.125 | 0.929 | 1.363 | 0.227 |
| QTc (/ms) | 1.000 | 0.998 | 1.002 | 0.931 |
| Female | 0.881 | 0.681 | 1.140 | 0.335 |
| IHD (vs DCM) | 1.320 | 1.074 | 1.623 | **0.008** |
| Secondary (vs primary) | 1.937 | 1.603 | 2.341 | **<0.001** |
| NYHA I | ref |  |  |  |
| NYHA II | 0.941 | 0.757 | 1.171 | 0.587 |
| NYHA III | 0.675 | 0.520 | 0.875 | **0.003** |
| NYHA IV | 1.430 | 0.455 | 4.494 | 0.541 |
| CRT-D (vs VVI/DDD) | 0.641 | 0.510 | 0.805 | **<0.001** |
| Stroke | 1.267 | 0.941 | 1.707 | 0.119 |
| DM | 1.091 | 0.862 | 1.381 | 0.469 |
| AF | 0.987 | 0.797 | 1.222 | 0.903 |
| AHT | 1.024 | 0.848 | 1.237 | 0.805 |
| BB | 0.643 | 0.513 | 0.807 | **<0.001** |
| ACE-I or ARB | 0.978 | 0.728 | 1.314 | 0.883 |
| Loop | 1.008 | 0.834 | 1.217 | 0.938 |
| Aldactone | 0.795 | 0.658 | 0.961 | **0.018** |
| Anti-aggregant | 1.146 | 0.939 | 1.400 | 0.180 |
| Anticoagulation | 0.952 | 0.774 | 1.170 | 0.638 |
| Amiodarone | 1.483 | 1.227 | 1.793 | **<0.001** |
| Sotalol | 0.799 | 0.257 | 2.489 | 0.699 |
| Digitalis | 1.663 | 1.245 | 2.222 | **0.001** |
| Statin | 0.800 | 0.662 | 0.967 | **0.021** |
| LBBB | 0.731 | 0.585 | 0.914 | **0.006** |
| Anterior fQRS | 0.887 | 0.553 | 1.422 | 0.618 |
| Lateral fQRS | 0.880 | 0.668 | 1.158 | 0.361 |
| Inferior fQRS | 0.984 | 0.809 | 1.197 | 0.874 |
| Any fQRS | 0.975 | 0.805 | 1.181 | 0.798 |

Age (years); body mass index, BMI (kg/m²); left ventricular ejection fraction, LVEF (%); creatinine (mg/dL); QTc interval, QTc (ms); ischemic heart disease, IHD; dilated cardiomyopathy, DCM; secondary vs primary prevention indication; New York Heart Association class, NYHA; diabetes mellitus, DM; atrial fibrillation, AF; arterial hypertension, AHT; betablocker, BB; angiotensin converting enzyme inhibitor, ACE-I; angiotensin receptor blocker, ARB; loop diuretic, loop; ever appropriate ICD therapy (anti-tachypacing or shock), ever appropriate; left bundle branch block, LBBB.

Table S6. Univariate Cox analysis regarding appropriate ICD shocks

| **APPROPRIATE SHOCK** |  | 95% confidence interval | |  |
| --- | --- | --- | --- | --- |
|  | HR | Lower | Upper | p-value |
| Age (/year) | 1.002 | 0.992 | 1.012 | 0.716 |
| BMI (/kg/m²) | 1.002 | 0.977 | 1.028 | 0.853 |
| LVEF (/%) | 1.006 | 0.997 | 1.014 | 0.216 |
| Creatinine (/mg/dL) | 1.221 | 0.993 | 1.501 | 0.058 |
| QTc (/ms) | 1.000 | 0.998 | 1.003 | 0.831 |
| Female | 0.794 | 0.584 | 1.078 | 0.139 |
| IHD (vs DCM) | 1.718 | 1.336 | 2.209 | **<0.001** |
| Secondary (vs primary) | 2.134 | 1.714 | 2.657 | **<0.001** |
| NYHA I | ref |  |  |  |
| NYHA II | 0.917 | 0.718 | 1.171 | 0.487 |
| NYHA III | 0.512 | 0.373 | 0.701 | **<0.001** |
| NYHA IV | 2.010 | 0.637 | 6.343 | 0.234 |
| CRT-D (vs VVI/DDD) | 0.495 | 0.373 | 0.658 | **<0.001** |
| Stroke | 0.973 | 0.668 | 1.418 | 0.888 |
| DM | 0.975 | 0.738 | 1.289 | 0.861 |
| AF | 1.053 | 0.825 | 1.344 | 0.677 |
| AHT | 1.059 | 0.852 | 1.317 | 0.603 |
| BB | 0.544 | 0.424 | 0.700 | **<0.001** |
| ACE-I or ARB | 0.835 | 0.603 | 1.155 | 0.276 |
| Loop | 1.046 | 0.842 | 1.300 | 0.683 |
| Aldactone | 0.708 | 0.568 | 0.882 | **0.002** |
| Anti-aggregant | 1.268 | 1.003 | 1.603 | **0.047** |
| Anticoagulation | 0.923 | 0.727 | 1.172 | 0.509 |
| Amiodarone | 1.643 | 1.321 | 2.043 | **<0.001** |
| Sotalol | 1.122 | 0.360 | 3.497 | 0.843 |
| Digitalis | 1.830 | 1.323 | 2.531 | **<0.001** |
| Statin | 0.721 | 0.580 | 0.896 | **0.003** |
| LBBB | 0.688 | 0.530 | 0.893 | **0.005** |
| Anterior fQRS | 1.003 | 0.597 | 1.685 | 0.990 |
| Lateral fQRS | 0.948 | 0.693 | 1.295 | 0.736 |
| Inferior fQRS | 1.043 | 0.833 | 1.305 | 0.713 |
| Any fQRS | 1.032 | 0.828 | 1.286 | 0.781 |

Age (years); body mass index, BMI (kg/m²); left ventricular ejection fraction, LVEF (%); creatinine (mg/dL); QTc interval, QTc (ms); ischemic heart disease, IHD; dilated cardiomyopathy, DCM; secondary vs primary prevention indication; New York Heart Association class, NYHA; diabetes mellitus, DM; atrial fibrillation, AF; arterial hypertension, AHT; betablocker, BB; angiotensin converting enzyme inhibitor, ACE-I; angiotensin receptor blocker, ARB; loop diuretic, loop; ever appropriate ICD therapy (anti-tachypacing or shock), ever appropriate; left bundle branch block, LBBB.

Table S7. Univariate Cox analysis regarding inappropriate ICD shocks

| **INAPPROPRIATE SHOCK** |  | 95% confidence interval | |  |
| --- | --- | --- | --- | --- |
|  | HR | Lower | Upper | p-value |
| Age (/year) | 0.980 | 0.968 | 0.993 | **0.002** |
| BMI (/kg/m²) | 0.966 | 0.930 | 1.004 | 0.079 |
| LVEF (/%) | 1.005 | 0.992 | 1.017 | 0.480 |
| Creatinine (/mg/dL) | 1.225 | 0.897 | 1.674 | 0.202 |
| QTc (/ms) | 0.998 | 0.994 | 1.001 | 0.246 |
| Female | 0.811 | 0.520 | 1.263 | 0.353 |
| IHD (vs DCM) | 1.022 | 0.731 | 1.428 | 0.900 |
| Secondary (vs primary) | 1.166 | 0.845 | 1.609 | 0.348 |
| NYHA I | ref |  |  |  |
| NYHA II | 0.849 | 0.585 | 1.230 | 0.386 |
| NYHA III | 0.888 | 0.585 | 1.346 | 0.575 |
| NYHA IV | 1.787 | 0.246 | 12.988 | 0.566 |
| CRT-D (vs VVI/DDD) | 0.844 | 0.582 | 1.224 | 0.371 |
| Stroke | 0.679 | 0.358 | 1.290 | 0.237 |
| DM | 0.840 | 0.547 | 1.291 | 0.428 |
| AF | 1.738 | 1.248 | 2.420 | **0.001** |
| AHT | 0.684 | 0.497 | 0.941 | **0.020** |
| BB | 0.900 | 0.600 | 1.350 | 0.612 |
| ACE-I or ARB | 0.611 | 0.400 | 0.934 | **0.023** |
| Loop | 1.100 | 0.800 | 1.511 | 0.558 |
| Aldactone | 0.808 | 0.585 | 1.114 | 0.193 |
| Anti-aggregant | 0.696 | 0.505 | 0.961 | **0.028** |
| Anticoagulation | 1.225 | 0.875 | 1.715 | 0.236 |
| Amiodarone | 1.216 | 0.882 | 1.675 | 0.232 |
| Sotalol | 0.791 | 0.111 | 5.657 | 0.815 |
| Digitalis | 1.539 | 0.941 | 2.518 | 0.086 |
| Statin | 0.681 | 0.495 | 0.935 | **0.018** |
| LBBB | 0.800 | 0.551 | 1.161 | 0.240 |
| Anterior fQRS | 1.059 | 0.496 | 2.263 | 0.882 |
| Lateral fQRS | 1.242 | 0.813 | 1.895 | 0.316 |
| Inferior fQRS | 0.817 | 0.582 | 1.147 | 0.243 |
| Any fQRS | 0.877 | 0.633 | 1.215 | 0.430 |

Age (years); body mass index, BMI (kg/m²); left ventricular ejection fraction, LVEF (%); creatinine (mg/dL); QTc interval, QTc (ms); ischemic heart disease, IHD; dilated cardiomyopathy, DCM; secondary vs primary prevention indication; New York Heart Association class, NYHA; diabetes mellitus, DM; atrial fibrillation, AF; arterial hypertension, AHT; betablocker, BB; angiotensin converting enzyme inhibitor, ACE-I; angiotensin receptor blocker, ARB; loop diuretic, loop; ever appropriate ICD therapy (anti-tachypacing or shock), ever appropriate; left bundle branch block, LBBB.

Table S8. Multivariable Cox analysis regarding appropriate ICD therapy

| **EVER APPROPRIATE** |  | 95% Confidence Interval | |  |
| --- | --- | --- | --- | --- |
|  | HR | Lower | Upper | p-value |
| IHD (vs DCM) | 1.284 | 0.985 | 1.673 | 0.064 |
| Secondary (vs primary) | 1.588 | 1.241 | 2.031 | **<0.001** |
| NYHA I | ref |  |  |  |
| II | 0.999 | 0.782 | 1.275 | 0.991 |
| III | 0.917 | 0.644 | 1.306 | 0.632 |
| IV | 2.646 | 0.794 | 8.820 | 0.113 |
| CRT-D (vs VVI/DDD) | 0.969 | 0.665 | 1.411 | 0.868 |
| BB | 0.792 | 0.608 | 1.031 | 0.083 |
| Aldactone | 1.030 | 0.822 | 1.291 | 0.795 |
| Amiodarone | 1.229 | 0.981 | 1.538 | 0.073 |
| Digitalis | 1.752 | 1.267 | 2.423 | **0.001** |
| Statin | 0.813 | 0.645 | 1.025 | 0.080 |
| LBBB | 0.895 | 0.670 | 1.197 | 0.455 |

Ischemic heart disease, IHD; dilated cardiomyopathy, DCM; secondary vs primary prevention indication; New York Heart Association class, NYHA; betablocker, BB; left bundle branch block, LBBB.

Table S9. Multivariable Cox analysis regarding appropriate ICD shock

| **APPROPRIATE SHOCK** |  | 95% Confidence Interval | |  |
| --- | --- | --- | --- | --- |
|  | HR | Lower | Upper | p-value |
| Creatinine (mg/dL) | 1.210 | 0.946 | 1.547 | 0.130 |
| IHD (vs DCM) | 1.698 | 1.200 | 2.402 | **0.003** |
| Secondary (vs primary) | 1.511 | 1.140 | 2.002 | **0.004** |
| NYHA I | ref |  |  |  |
| II | 1.007 | 0.765 | 1.325 | 0.960 |
| III | 0.780 | 0.508 | 1.197 | 0.255 |
| IV | 3.913 | 1.124 | 13.624 | **0.032** |
| CRT-D (vs VVI/DDD) | 0.687 | 0.435 | 1.085 | 0.107 |
| BB | 0.747 | 0.557 | 1.003 | 0.052 |
| Aldactone | 0.990 | 0.761 | 1.287 | 0.938 |
| Anti-aggregant | 1.054 | 0.783 | 1.420 | 0.728 |
| Amiodarone | 1.252 | 0.965 | 1.625 | 0.091 |
| Digitalis | 2.095 | 1.450 | 3.027 | **<0.001** |
| Statin | 0.655 | 0.503 | 0.854 | **0.002** |
| LBBB | 1.117 | 0.805 | 1.551 | 0.507 |

Creatinine (mg/dL); ischemic heart disease, IHD; dilated cardiomyopathy, DCM; secondary vs primary prevention indication; New York Heart Association class, NYHA; betablocker, BB; left bundle branch block, LBBB.

Table S10. Multivariable Cox analysis regarding inappropriate ICD therapy.

| **INAPPROPRIATE SHOCK** |  | 95% Confidence Interval | |  |
| --- | --- | --- | --- | --- |
|  | HR | Lower | Upper | p-value |
| Age (/year) | 0.981 | 0.967 | 0.995 | **0.010** |
| BMI (/kg/m²) | 0.978 | 0.941 | 1.017 | 0.270 |
| AF | 1.992 | 1.381 | 2.875 | **<0.001** |
| AHT | 0.840 | 0.589 | 1.198 | 0.335 |
| ACE-I/ARB | 0.689 | 0.443 | 1.072 | 0.098 |
| Anti-aggregant | 1.009 | 0.701 | 1.450 | 0.963 |
| Digitalis | 1.201 | 0.713 | 2.023 | 0.490 |
| Statin | 0.856 | 0.601 | 1.221 | 0.391 |

Body mass index, BMI (kg/m²); atrial fibrillation, AF; arterial hypertension, AHT; angiotensin converting enzyme inhibitor, ACE-I; angiotensin receptor blocker, ARB.

Table S11. Baseline patient characteristics by fQRS for primary prevention subgroup

| **QRS fragmentation** | Overall |  | fQRS (-) |  | fQRS (+) |  | p-value |
| --- | --- | --- | --- | --- | --- | --- | --- |
| N=792 | Mean | SD | Mean | SD | Mean | SD |  |
| Age (years) | 61.5 | 11.6 | 62.2 | 10.4 | 60.1 | 13.1 | 0.224 |
| BMI (kg/m²) | 26.6 | 4.5 | 26.7 | 4.7 | 26.6 | 4.3 | 0.923 |
| LVEF (%) | 27 | 9 | 27 | 9 | 27 | 9 | 0.931 |
| Creatinine (mg/dL) | 1.23 | 0.46 | 1.24 | 0.05 | 1.22 | 0.43 | 0.336 |
| QTc (ms) | 439 | 49 | 439 | 47 | 440 | 51 | 0.756 |
|  | Number | % | Number | % | Number | % |  |
| Female | 158 | 19.9 | 83 | 18.5 | 75 | 21.8 | 0.253 |
| IHD (vs DCM) | 435 | 54.9 | 252 | 56.3 | 183 | 53.2 | 0.392 |
| NYHA I | 156 | 19.7 | 99 | 22.1 | 57 | 16.6 | 0.121 |
| II | 351 | 44.3 | 201 | 44.9 | 150 | 43.6 |  |
| III | 278 | 35.1 | 145 | 32.4 | 133 | 38.7 |  |
| IV | 7 | 0.9 | 3 | 0.7 | 4 | 1.2 |  |
| CRT-D (vs VVI/DDD) | 322 | 40.7 | 162 | 36.2 | 160 | 46.6 | **0.003** |
| Stroke | 78 | 9.8 | 40 | 8.9 | 38 | 11.0 | 0.321 |
| DM | 181 | 22.9 | 99 | 22.1 | 82 | 23.8 | 0.563 |
| AF | 214 | 27.1 | 115 | 25.7 | 99 | 28.9 | 0.316 |
| AHT | 403 | 50.9 | 234 | 52.2 | 169 | 49.1 | 0.386 |
| BB | 719 | 90.8 | 402 | 89.7 | 317 | 92.2 | 0.243 |
| ACE-I or ARB | 76 | 90.4 | 409 | 91.3 | 307 | 89.2 | 0.331 |
| Loop | 438 | 55.3 | 238 | 53.1 | 200 | 58.1 | 0.159 |
| Aldactone | 518 | 65.4 | 285 | 63.6 | 233 | 67.7 | 0.227 |
| Anti-aggregant | 423 | 53.4 | 251 | 56.0 | 172 | 50.0 | 0.092 |
| Anticoagulation | 285 | 36.0 | 156 | 34.8 | 129 | 37.5 | 0.436 |
| Amiodarone | 179 | 22.6 | 99 | 22.1 | 80 | 23.3 | 0.699 |
| Sotalol | 8 | 1.0 | 5 | 1.1 | 3 | 0.9 | 0.734 |
| Digitalis | 71 | 9.0 | 39 | 8.7 | 32 | 9.3 | 0.771 |
| Statin | 516 | 65.2 | 295 | 65.8 | 221 | 64.2 | 0.639 |
| Ever appropriate | 207 | 26.1 | 128 | 28.6 | 79 | 23.0 | 0.075 |
| LBBB | 296 | 41.9 | 147 | 37.3 | 149 | 47.8 | **0.005** |

Age (years); body mass index, BMI (kg/m²); left ventricular ejection fraction, LVEF (%); creatinine (mg/dL); QTc interval, QTc (ms); ischemic heart disease, IHD; dilated cardiomyopathy, DCM; secondary vs primary prevention indication; New York Heart Association class, NYHA; diabetes mellitus, DM; atrial fibrillation, AF; arterial hypertension, AHT; betablocker, BB; angiotensin converting enzyme inhibitor, ACE-I; angiotensin receptor blocker, ARB; loop diuretic, loop; ever appropriate ICD therapy (anti-tachypacing or shock), ever appropriate; left bundle branch block, LBBB.
